# Effects of alfalfa saponins on the production performance, serum biochemical factors, and immune factors in Small-Tailed Han sheep

**DOI:** 10.3389/fvets.2022.924373

**Published:** 2022-07-22

**Authors:** Fan Yang, Fang Yang, Zhen-Han Zhai, Sai-Qiao Wang, Lu Zhao, Bing-Lei Zhang, Jun-Cheng Chen, Yu-Qin Wang

**Affiliations:** College of Animal Science and Technology, Henan University of Science and Technology, Luoyang, China

**Keywords:** alfalfa saponins, Small-Tailed Han sheep, production performance, serum biochemical factors, immune factors

## Abstract

This study aimed to determine the potential effects of alfalfa saponins on the production performance, serum biochemical factors, and immune factors in sheep. Twenty Small-Tailed Han sheep were equally and randomly divided into Groups 1–4, fed with diets containing 0, 5, 10, and 20 g alfalfa saponins per kg, respectively, for 40 consecutive days. During the treatments, the body weight change was recorded for each sheep. Before, during, and after the treatments of alfalfa saponins, serum was collected from each group to compare the levels of biochemical and immune factors. All sheep were killed after the treatments, and the longissimus dorsi muscle was collected to compare the meat quality. The results validated the effects of alfalfa saponins on the growth performance and meat quality in Small-Tailed Han sheep, and the supplementation level of 10 g/kg was the best. Alfalfa saponins also had effects on the levels of biochemical factors in serum. However, both dose- and time-dependent effects were observed. After a shorter feeding period (14 days), the concentrations of cholesterol (CHOL) and low-density lipoprotein (LDL) in Groups 2, 3, and 4 were all lower than those in the control group; however, when alfalfa saponins were continuously fed, this effect was not apparent or even gone. Supplying alfalfa saponins increased serum concentrations of IgA, IgG, IgE, IgM, IL-1, IFN-α, and IFN-β. And this effect was distinctly observed in Groups 3 and 4. Based on the current results, the alfalfa saponins concentration of 10 g/kg (for 14 consecutive days) could be suggested as the optimum ratio for good health conditions of Small-Tailed Han sheep.

## Introduction

Alfalfa (*Medicago sativa*) is cultivated worldwide as an essential legume for grazing, hay, silage, green manure, and cover crop (1). It contains characteristic secondary metabolites such as saponins, coumarins, isoflavones, and alkaloids. Among them, alfalfa saponins have been shown to have various biological and pharmaceutical functions. Their lipid-soluble core and steroid or triterpene structures confer the membrane-solubilizing activity of alfalfa saponins and explain their antibacterial, antitumor, and anti-inflammatory properties in animals (2–4). Alfalfa saponins can act on cholesterol and regulate lipid metabolism (5, 6). Due to their excellent antioxidant properties, alfalfa saponins have been shown to excel in promoting growth characteristics in weaned piglets (4).

These various biological and pharmaceutical functions of alfalfa saponins have been demonstrated in different animal species, including ruminants (5, 7–9), pigs (4), poultry (10, 11), and aquaculture species (12). However, there are few reports about their applications in Small-tailed Han sheep, a well-known local sheep breed in China. Farmers favor the Small-tailed Han sheep because of their rapid growth and development, high reproductive performance, and strong adaptability (13). To our knowledge, this breed has become one of the most commonly farmed sheep species in China. With the rapid development of intensive sheep farming, however, Small-Tailed Han sheep are suffering from many pathogens because of the high breeding density, which has caused substantial economic losses. It has been demonstrated that the lipid-soluble extract from alfalfa, including alfalfa saponins, could modulate the host immunity in mice infected with *Citrobacter rodentium* (14). Similar immunity-enhancing activity has also been found for alfalfa saponins in the crossbred lambs (15). However, it is unknown whether there is a similar immune-enhancing effect in the Small-Tailed Han sheep. Therefore, the present study aimed to investigate the potential effects of alfalfa saponins on the production performance, serum biochemical indexes, and immune function in the Small-Tailed Han sheep.

## Materials and methods

### Chemical reagents

The alfalfa saponins extract (Lot No. 201405102) with a purity of 518.5 g/kg dry matter was purchased from Xi'an Realin Biotechnology Co., Ltd. (Xi'an, Shaanxi, China). This commercial product was extracted from the leaves and roots of alfalfa (*Medicago sativa*), purified through high-performance liquid chromatography, and quantified with oleanolic acid as the standard substance (5).

### Animals and experimental design

Animal experiments were conducted on the sheep farm of Xianda Animal Husbandry Development Co., Ltd. (Luoyang, Henan, China) under the protocols approved by the Institutional Animal Care and Use Committee (IACUC) of Henan University of Science and Technology. Twenty healthy male Small-Tailed Han sheep were selected, with an average body weight of 36.08 ± 2.68 kg. Five sheep were randomly kept in pen (5 m × 8 m) equipped with a bamboo slatted floor and water taps. All sheep were acclimated for at least 10 days and fed commercial feed twice daily in the morning and evening, supplemented with silage corn stover at other times and water *ad libitum*.

The sheep in each pen were grouped as Groups 1, 2, 3, and 4, respectively. Group 1 (Control group) received the basal diet (Table 1), and Groups 2, 3, and 4 received different doses of alfalfa saponins extract powder which was mixed into the basal diet (5, 10, 20 g/kg basal diet, respectively). All sheep were fed the corresponding diets for 40 days.

**Table 1:** Ingredients and chemical composition of the basal diet.

**Items**	**Diets**
**Ingredient (g/kg fed basis)**
Corn silage	200
Peanut vine	300
Corn	290
Soybean meal	140
Wheat bran	35
Salt	5
Sodium bicarbonate	5
Minerals and vitamin premix^a^	25
**Chemical composition (g/kg dry matter basis)**
Dry matter (g/kg fresh basis)	850
Crude protein	160
Neutral detergent fiber	265
Acid detergent fiber	183
Metabolizable energy (MJ/kg)	10.5
Calcium	5.3
Phosphorus	2.6

*^a^ Premix (/kg): Cu, 0.3 g; Fe, 1.6 g; Mn, 1.8 mg; Se, 0.6 mg; Zn, 260 mg; Vitamin A, 22,000 IU; Vitamin D3, 70,000 IU*.

### Production performance

#### Growth performance

The entire feeding period lasted 40 days. Before alfalfa saponins treatments, the fasting body weight of each sheep was weighed and recorded as the initial weight. Then the body weight was recorded prior to the morning feeding on days 8, 15, 25, 35, and 41 days. The initial and final body weights were recorded. Average daily gain (ADG, kg/day) was calculated between weighing intervals.

#### Meat quality

After the 40-day feeding period, sheep in each group were slaughtered. Approximately 200 g of the longissimus dorsi muscle was collected to determine meat quality traits, including cooking loss, drip loss, and shear force. For cooking loss determination, a meat sample with 2.5 cm thickness was heated in an 80°C water bath for 30 min. The proportion of weight lost during heating is the cooking loss (%). Re-weighting was done when the meat cooled down for 20 min at room temperature. For the drip loss determination, a 2.0 cm thick meat sample was weighed and then hung in a refrigerator at 4°C. The meat sample was weighed again after 48 h. The proportion of weight loss was determined as the drip loss. The shear force was determined by a digital texture analyzer (C-LM3B; Beijing Tianxiang Feiyu Instrument & Equipment Co., Ltd.).

### Serum biochemical factors and immune factors

Approximately 10 ml of blood samples were collected from sheep before morning feeding on days 0 (before treatment), 15, 25, and 41 (before the slaughter). After the collection, blood samples were quickly stored at 4°C for 3 h, followed by centrifugation at 2,000 g for 10 min. Then the serum was collected and stored at −20°C for further determination of biochemical and immune factors. The automatic biochemical analyzer AU5400 (Beckman Coulter Commercial Enterprise, Shanghai, China) was used to determine the levels of serum biochemical factors, including alanine transaminase (ALT), aspartate aminotransferase (AST), total protein (TP), albumin (ALB), globulin (GLO), Urea-N, creatinine (CRT), glucose (GLU), calcium, phosphorus, triglyceride (TRIG), cholesterol (CHOL), high-density lipoprotein (HDL), low-density lipoprotein (LDL), creatine kinase (CK), lactate dehydrogenase (LDH), glutamyltransferase (GT), and alkaline phosphatase (ALP). Based on our previous studies (16, 17), the commercial enzyme-linked immunosorbent assay (ELISA) kits (Nanjing Jiancheng Bioengineering Institute, Jiangsu, China) were used to determine immunoglobulins, interleukins, and interferons levels, including IgA, IgG, IgE, IgM, IL-1, IL-6, IFN-α, IFN-β, and IFN-γ.

### Statistical analysis

All data were expressed as mean ± SE (standard error) or SD (standard deviation). Normality and homogeneity of variance were checked with the Kolmogorov-Smirnov test. Statistical analysis was performed using SPSS 17.0 software (SPSS Inc., Chicago, IL, USA). A two-way analysis of variance (two-way ANOVA) was used for serum biochemical factors to evaluate the interaction between feeding periods and doses. And least significant difference (LSD) test was used to compare the differences between the four groups or periods. Differences in the other parameters were processed by one-way analysis of variance (ANOVA) followed by Duncan's multiple range test, considering *P* < 0.05 as the significance level.

## Results

### Growth performance

There were differences, but not significant, in the body weights of the four groups before alfalfa saponins treatments. After different treatment periods, the body weight changes in each group are shown in Table 2. After 7 and 14 days of continuous feeding, the average body weight of Small-Tailed Han sheep in the three treatment groups was significantly higher than that of the control group (*P* < 0.05). However, at the last three weighing points, the average body weight in Group 3 was significantly higher than those in Groups 1 and 4 (*P* < 0.05). After different feeding periods, the average daily gain in each group was also compared in Table 2. During the first four feeding periods, the ADGs in the three treatment groups were significantly higher than those in the control group (*P* < 0.05). However, Group 3 had the best ADG considering the 40-day feeding period, which was significantly higher than Groups 1 and 4 (*P* < 0.05), indicating that the supplementation level of 10 g/kg was the best for growth performance.

**Table 2:** Effects of alfalfa saponins on growth performances (mean ± SD) of Small-Tailed Han sheep.

**Items**	**Group 1**	**Group 2**	**Group 3**	**Group 4**
Initial BW (kg) Day 0	32.9 ± 2.3	35.8 ± 2.6	36.3 ± 1.2	35.7 ± 2.2
**Final BW (kg)**
Day 8	33.1 ± 2.0^b^	37.4 ± 3.0^a^	39.4 ± 2.0^a^	36.4 ± 2.2^a^
Day 15	33.6 ± 2.6^b^	38.6 ± 2.8^a^	40.9 ± 2.2^a^	37.3 ± 3.1^a^
Day 25	34.3 ± 2.5^c^	40.7 ± 2.3^ab^	42.8 ± 2.9^a^	38.8 ± 3.3^b^
Day 35	34.3 ± 2.7^c^	42.4 ± 2.2^ab^	44.2 ± 2.8^a^	39.8 ± 3.5^b^
Day 41	36.5 ± 1.9^c^	44.2 ± 2.6^ab^	46.6 ± 3.3^a^	42.4 ± 2.7^b^
**ADG (kg/day)**
0–7 days	0.029 ± 0.004^c^	0.229 ± 0.008^a^	0.443 ± 0.021^a^	0.100 ± 0.015^b^
8–14 days	0.071 ± 0.011^c^	0.171 ± 0.014^a^	0.214 ± 0.017^a^	0.129 ± 0.013^ab^
15–24 days	0.070 ± 0.008^b^	0.210 ± 0.011^a^	0.190 ± 0.016^a^	0.150 ± 0.018^a^
25–34 days	0.040 ± 0.003^b^	0.170 ± 0.019^a^	0.140 ± 0.009^a^	0.100 ± 0.008^a^
35–40 days	0.300 ± 0.014^b^	0.300 ± 0.016^b^	0.400 ± 0.016^a^	0.433 ± 0.026^a^
0–40 days	0.090 ± 0.031^c^	0.210 ± 0.024^ab^	0.258 ± 0.071^a^	0.168 ± 0.073^b^

*Data in a row with different small letters represented a significant difference at a 5% level, while the same letters indicated no significant difference*.

### Meat quality

After 40 days of treatment with alfalfa saponins, all sheep were slaughtered, and their longissimus dorsi muscles were collected for meat quality comparison. The results are shown in Table 3. The drip loss in the three treatment groups decreased compared to the control group, but without a significant difference. The lowest cooking loss (31.74%) was observed in Group 3 (*P* < 0.05). Regarding shear force, Group 3 also had the smallest values (2.95 ± 0.58 kgF), significantly different from Groups 1 and 4, indicating that the supplementation of alfalfa saponins at 10 g/kg might be the best one.

**Table 3:** Comparison of meat qualities (mean ± SD) among four groups.

**Groups**	**Drip loss (%)**	**Cooking loss (%)**	**Shear force (kgF)**
Group 1	45.31 ± 4.76^a^	32.65 ± 4.61^a^	3.26 ± 0.57^a^
Group 2	44.30 ± 3.14^a^	32.84 ± 2.66^a^	3.06 ± 0.57^b^
Group 3	43.87 ± 2.78^a^	31.74 ± 2.76^b^	2.95 ± 0.58^b^
Group 4	44.18 ± 2.02^a^	34.48 ± 5.62^a^	3.19 ± 0.52^a^

*Data in a column with different small letters represented a significant difference at a 5% level, while the same letters indicated no significant difference*.

### Serum biochemical factors

No interaction between feeding time and feeding dose was observed for all serum biochemical factors after the two-way ANOVA. Before treatments with alfalfa saponins, no significant differences were found in the serum biochemical factors among the four groups (Table 4). After 14, 24, and 40 days of continuous feeding, the changes among different treatment groups were observed (Table 4). In addition to the comparison of dose-dependent effect, the time-dependent effects were also compared for the 4 groups (Table 5).

**Table 4:** Comparison of serum biochemical parameter levels (mean ± SE) among four treatment groups (n = 5 in each group) after 0 (before feeding), 14, 24, and 40 consecutive days of feeding.

**Continuous feeding time (days)**	**Biochemical parameter**	**Unit**	**Group 1**	**Group 2**	**Group 3**	**Group 4**
0	ALT	U/L	11.60 ± 3.26	13.00 ± 0.89	11.00 ± 2.07	16.60 ± 2.25
	AST	U/L	93.00 ± 4.95	91.20 ± 4.97	98.60 ± 6.44	95.20 ± 4.22
	TP	g/L	63.36 ± 1.06	62.96 ± 1.40	64.72 ± 1.33	65.22 ± 1.76
	ALB	g/L	21.32 ± 0.78	21.28 ± 0.55	21.50 ± 1.06	22.16 ± 0.47
	GLO	g/L	42.04 ± 0.68	41.68 ± 1.45	43.22 ± 1.36	43.06 ± 2.12
	Urea-N	mmol/L	5.91 ± 0.36	6.35 ± 0.39	7.04 ± 0.44	5.45 ± 0.42
	CRT	mmol/L	41.20 ± 3.28	40.80 ± 2.58	43.60 ± 1.75	40.80 ± 2.06
	GLU	mmol/L	3.49 ± 0.11	3.32 ± 0.15	3.07 ± 0.20	3.13 ± 0.23
	Calcium	mmol/L	2.42 ± 0.05	2.44 ± 0.08	2.50 ± 0.05	2.51 ± 0.05
	Phosphorus	mmol/L	2.72 ± 0.15	2.30 ± 0.18	2.31 ± 0.12	2.50 ± 0.24
	TRIG	mmol/L	0.48 ± 0.04	0.54 ± 0.14	0.38 ± 0.06	0.31 ± 0.04
	CHOL	mmol/L	1.77 ± 0.08	1.67 ± 0.04	1.89 ± 0.20	1.69 ± 0.03
	HDL	mmol/L	0.91 ± 0.05	0.83 ± 0.03	0.93 ± 0.09	0.86 ± 0.03
	LDL	mmol/L	0.46 ± 0.03	0.49 ± 0.06	0.56 ± 0.07	0.49 ± 0.01
	CK	U/L	164.20 ± 12.10	224.40 ± 32.31	248.80 ± 32.32	257.80 ± 37.83
	LDH	U/L	269.00 ± 13.79	311.60 ± 22.10	322.60 ± 11.34	273.60 ± 12.36
	GT	U/L	54.80 ± 5.34	63.60 ± 6.77	70.80 ± 7.36	61.20 ± 6.76
	ALP	U/L	185.40 ± 11.87	209.00 ± 30.36	174.40 ± 16.98	196.80 ± 43.42
14	ALT	U/L	15.60 ± 3.20	13.40 ± 1.21	12.00 ± 2.07	17.60 ± 1.21
	AST	U/L	90.00 ± 8.63	88.80 ± 4.71	96.80 ± 5.86	92.00 ± 6.99
	TP	g/L	66.92 ± 1.97^b^	66.70 ± 1.50^b^	69.08 ± 1.26^ab^	74.72 ± 2.67^a^
	ALB	g/L	21.74 ± 1.15	22.76 ± 0.47	23.70 ± 0.96	22.92 ± 1.02
	GLO	g/L	45.18 ± 2.17	43.94 ± 1.39	45.38 ± 1.88	51.80 ± 3.44
	Urea-N	mmol/L	6.18 ± 0.22	6.26 ± 0.57	6.71 ± 0.25	6.05 ± 0.28
	CRT	mmol/L	51.40 ± 3.31	45.80 ± 5.02	49.40 ± 1.44	43.00 ± 2.65
	GLU	mmol/L	3.34 ± 0.10	3.28 ± 0.16	3.34 ± 0.29	3.57 ± 0.27
	Calcium	mmol/L	2.45 ± 0.09	2.51 ± 0.04	2.58 ± 0.04	2.56 ± 0.07
	Phosphorus	mmol/L	2.85 ± 0.45	3.00 ± 0.17	3.06 ± 0.21	3.16 ± 0.16
	TRIG	mmol/L	0.60 ± 0.06	0.37 ± 0.09	0.49 ± 0.09	0.44 ± 0.06
	CHOL	mmol/L	2.18 ± 0.19^a^	1.81 ± 0.11^ab^	1.65 ± 0.08^b^	1.80 ± 0.09^b^
	HDL	mmol/L	0.90 ± 0.04	0.97 ± 0.07	1.09 ± 0.10	0.91 ± 0.05
	LDL	mmol/L	0.62 ± 0.04^a^	0.45 ± 0.03^b^	0.43 ± 0.04^b^	0.50 ± 0.03^b^
	CK	U/L	136.60 ± 4.07	188.20 ± 19.58	155.60 ± 24.76	197.40 ± 19.11
	LDH	U/L	288.00 ± 38.35	292.80 ± 17.66	328.20 ± 28.79	306.40 ± 20.60
	GT	U/L	62.40 ± 2.34	71.00 ± 6.86	75.60 ± 7.91	63.20 ± 5.78
	ALP	U/L	218.40 ± 40.91	256.80 ± 44.29	297.60 ± 29.78	282.80 ± 40.26
24	ALT	U/L	13.20 ± 2.75	13.00 ± 1.55	11.80 ± 1.85	17.20 ± 0.58
	AST	U/L	88.40 ± 5.32	92.20 ± 5.54	92.20 ± 4.89	96.60 ± 8.05
	TP	g/L	66.64 ± 2.08^b^	66.32 ± 1.84^b^	68.84 ± 0.97^ab^	74.44 ± 2.42^a^
	ALB	g/L	21.90 ± 1.09	23.34 ± 0.43	23.68 ± 0.92	23.52 ± 0.93
	GLO	g/L	44.74 ± 2.09	42.98 ± 1.93	45.16 ± 1.58	50.92 ± 3.32
	Urea-N	mmol/L	5.10 ± 0.29	5.41 ± 0.48	6.43 ± 0.48	5.82 ± 0.30
	CRT	mmol/L	48.80 ± 2.58	44.80 ± 3.81	46.80 ± 1.32	45.20 ± 2.42
	GLU	mmol/L	2.92 ± 0.20	3.05 ± 0.14	2.87 ± 0.33	3.34 ± 0.23
	Calcium	mmol/L	2.53 ± 0.07	2.53 ± 0.03	2.51 ± 0.03	2.51 ± 0.05
	Phosphorus	mmol/L	2.79 ± 0.22	2.88 ± 0.13	2.69 ± 0.20	2.89 ± 0.14
	TRIG	mmol/L	0.67 ± 0.10	0.73 ± 0.17	0.46 ± 0.07	0.70 ± 0.11
	CHOL	mmol/L	1.61 ± 0.07	1.88 ± 0.10	1.96 ± 0.16	1.81 ± 0.10
	HDL	mmol/L	0.88 ± 0.04	0.96 ± 0.07	1.03 ± 0.10	0.90 ± 0.05
	LDL	mmol/L	0.40 ± 0.03	0.50 ± 0.04	0.49 ± 0.03	0.50 ± 0.05
	CK	U/L	153.60 ± 11.17	144.40 ± 16.34	165.40 ± 15.23	203.20 ± 23.26
	LDH	U/L	268.00 ± 16.84	301.60 ± 18.15	292.60 ± 8.62	318.20 ± 19.91
	GT	U/L	60.00 ± 2.68	65.40 ± 6.11	65.80 ± 5.37	60.60 ± 3.72
	ALP	U/L	252.60 ± 51.75	294.40 ± 46.71	262.40 ± 34.14	287.00 ± 39.51
40	ALT	U/L	21.00 ± 5.13^a^	13.25 ± 1.38^ab^	11.20 ± 2.63^b^	20.80 ± 1.85^a^
	AST	U/L	94.67 ± 1.20	96.25 ± 7.82	100.60 ± 5.00	106.20 ± 11.27
	TP	g/L	68.73 ± 0.72	68.40 ± 2.13	68.12 ± 0.96	72.58 ± 2.88
	ALB	g/L	23.40 ± 1.47	24.15 ± 0.51	24.26 ± 0.53	24.22 ± 0.97
	GLO	g/L	45.33 ± 2.06	44.25 ± 2.48	43.86 ± 1.35	48.36 ± 3.09
	Urea-N	mmol/L	3.84 ± 0.60	2.92 ± 0.36	3.09 ± 0.37	3.29 ± 0.39
	CRT	mmol/L	44.67 ± 0.88	48.00 ± 4.10	50.40 ± 1.86	48.00 ± 1.97
	GLU	mmol/L	4.03 ± 0.20	4.09 ± 0.18	3.45 ± 0.20	4.48 ± 0.43
	Calcium	mmol/L	2.45 ± 0.18	2.55 ± 0.04	2.47 ± 0.05	2.49 ± 0.05
	Phosphorus	mmol/L	2.17 ± 0.23	2.71 ± 0.30	2.61 ± 0.23	2.52 ± 0.17
	TRIG	mmol/L	0.55 ± 0.04	0.56 ± 0.12	0.41 ± 0.06	0.49 ± 0.06
	CHOL	mmol/L	1.91 ± 0.13	2.04 ± 0.12	2.06 ± 0.21	1.96 ± 0.08
	HDL	mmol/L	0.91 ± 0.09	1.01 ± 0.05	0.96 ± 0.11	0.90 ± 0.05
	LDL	mmol/L	0.53 ± 0.03	0.62 ± 0.05	0.63 ± 0.05	0.65 ± 0.06
	CK	U/L	217.33 ± 40.33	125.00 ± 18.08	216.20 ± 54.06	230.40 ± 50.55
	LDH	U/L	363.00 ± 30.83	338.75 ± 31.65	331.8012.94	312.40 ± 42.69
	GT	U/L	49.00 ± 1.53	64.00 ± 8.88	58.603.63	57.00 ± 4.64
	ALP	U/L	295.33 ± 84.37	299.50 ± 46.80	321.4045.90	343.40 ± 55.14

*Data in a row with different small letters represented a significant difference at a 5% level, while the same or no letters indicated no significant difference*.

**Table 5:** Comparisons of serum biochemical parameter levels (mean ± SE) at different sampling time points in four groups (*n* = 5).

**Treatment groups**	**Biochemical parameter**	**Unit**	**Successive feeding for 7 days**	**Successive feeding for 14 days**	**Successive feeding for 24 days**	**Successive feeding for 40 days**
Group 1	ALT	U/L	11.60 ± 3.26	15.60 ± 3.20	13.20 ± 2.75	21.00 ± 5.13
	AST	U/L	93.00 ± 4.95	90.00 ± 8.63	88.40 ± 5.32	94.67 ± 1.20
	TP	g/L	63.36 ± 1.06	66.92 ± 1.97	66.64 ± 2.08	68.73 ± 0.72
	ALB	g/L	21.32 ± 0.78	21.74 ± 1.15	21.90 ± 1.09	23.40 ± 1.47
	GLO	g/L	42.04 ± 0.68	45.18 ± 2.17	44.74 ± 2.09	45.33 ± 2.06
	Urea-N	mmol/L	5.91 ± 0.36^ab^	6.18 ± 0.22^a^	5.10 ± 0.29^b^	3.84 ± 0.60^c^
	CRT	mmol/L	41.20 ± 3.28	51.40 ± 3.31	48.80 ± 2.58	44.67 ± 0.88
	GLU	mmol/L	3.49 ± 0.11^b^	3.34 ± 0.10^b^	2.92 ± 0.20^c^	4.03 ± 0.20^a^
	Calcium	mmol/L	2.42 ± 0.05	2.45 ± 0.09	2.53 ± 0.07	2.45 ± 0.18
	Phosphorus	mmol/L	2.72 ± 0.15	2.85 ± 0.45	2.79 ± 0.22	2.17 ± 0.23
	TRIG	mmol/L	0.48 ± 0.04	0.60 ± 0.06	0.67 ± 0.10	0.55 ± 0.04
	CHOL	mmol/L	1.77 ± 0.08	2.18 ± 0.19	1.61 ± 0.07	1.91 ± 0.13
	HDL	mmol/L	0.91 ± 0.05	0.90 ± 0.04	0.88 ± 0.04	0.91 ± 0.09
	LDL	mmol/L	0.46 ± 0.03	0.62 ± 0.04	0.40 ± 0.03	0.53 ± 0.03
	CK	U/L	164.20 ± 12.10^b^	136.60 ± 4.07^b^	153.60 ± 11.17^b^	217.33 ± 40.33^a^
	LDH	U/L	269.00 ± 13.79^b^	288.00 ± 38.35^ab^	268.00 ± 16.84^b^	363.00 ± 30.83^a^
	GT	U/L	54.80 ± 5.34	62.40 ± 2.34	60.00 ± 2.68	49.00 ± 1.53
	ALP	U/L	185.40 ± 11.87	218.40 ± 40.91	252.60 ± 51.75	295.33 ± 84.37
Group 2	ALT	U/L	13.00 ± 0.89	13.40 ± 1.21	13.00 ± 1.55	13.25 ± 1.38
	AST	U/L	91.20 ± 4.97	88.80 ± 4.71	92.20 ± 5.54	96.25 ± 7.82
	TP	g/L	62.96 ± 1.40	66.70 ± 1.50	66.32 ± 1.84	68.40 ± 2.13
	ALB	g/L	21.28 ± 0.55^c^	22.76 ± 0.47^b^	23.34 ± 0.43^ab^	24.15 ± 0.51^a^
	GLO	g/L	41.68 ± 1.45	43.94 ± 1.39	42.98 ± 1.93	44.25 ± 2.48
	Urea-N	mmol/L	6.35 ± 0.39^a^	6.26 ± 0.57^a^	5.41 ± 0.48^a^	2.92 ± 0.36^b^
	CRT	mmol/L	40.80 ± 2.58	45.80 ± 5.02	44.80 ± 3.81	48.00 ± 4.10
	GLU	mmol/L	3.32 ± 0.15^b^	3.28 ± 0.16^b^	3.05 ± 0.14^b^	4.09 ± 0.18^a^
	Calcium	mmol/L	2.44 ± 0.08	2.51 ± 0.04	2.53 ± 0.03	2.55 ± 0.04
	Phosphorus	mmol/L	2.30 ± 0.18	3.00 ± 0.17	2.88 ± 0.13	2.71 ± 0.30
	TRIG	mmol/L	0.54 ± 0.14	0.37 ± 0.09	0.73 ± 0.17	0.56 ± 0.12
	CHOL	mmol/L	1.67 ± 0.04^b^	1.81 ± 0.11^ab^	1.88 ± 0.10^ab^	2.04 ± 0.12^a^
	HDL	mmol/L	0.83 ± 0.03	0.97 ± 0.07	0.96 ± 0.07	1.01 ± 0.05
	LDL	mmol/L	0.49 ± 0.06^b^	0.45 ± 0.03^b^	0.50 ± 0.04^ab^	0.62 ± 0.05^a^
	CK	U/L	224.40 ± 32.31^a^	188.20 ± 19.58^ab^	144.40 ± 16.34^b^	125.00 ± 18.08^b^
	LDH	U/L	311.60 ± 22.10	292.80 ± 17.66	301.60 ± 18.15	338.75 ± 31.65
	GT	U/L	63.60 ± 6.77	71.00 ± 6.86	65.40 ± 6.11	64.00 ± 8.88
	ALP	U/L	209.00 ± 30.36	256.80 ± 44.29	294.40 ± 46.71	299.50 ± 46.80
Group 3	ALT	U/L	11.00 ± 2.07	12.00 ± 2.07	11.80 ± 1.85	11.20 ± 2.63
	AST	U/L	98.60 ± 6.44	96.80 ± 5.86	92.20 ± 4.89	100.60 ± 5.00
	TP	g/L	64.72 ± 1.33	69.08 ± 1.26	68.84 ± 0.97	68.12 ± 0.96
	ALB	g/L	21.50 ± 1.06	23.70 ± 0.96	23.68 ± 0.92	24.26 ± 0.53
	GLO	g/L	43.22 ± 1.36	45.38 ± 1.88	45.16 ± 1.58	43.86 ± 1.35
	Urea-N	mmol/L	7.04 ± 0.44^a^	6.71 ± 0.25^a^	6.43 ± 0.48^a^	3.09 ± 0.37^b^
	CRT	mmol/L	43.60 ± 1.75^b^	49.40 ± 1.44^a^	46.80 ± 1.32^ab^	50.40 ± 1.86^a^
	GLU	mmol/L	3.07 ± 0.20	3.34 ± 0.29	2.87 ± 0.33	3.45 ± 0.20
	Calcium	mmol/L	2.50 ± 0.05	2.58 ± 0.04	2.51 ± 0.03	2.47 ± 0.05
	Phosphorus	mmol/L	2.31 ± 0.12	3.06 ± 0.21	2.69 ± 0.20	2.61 ± 0.23
	TRIG	mmol/L	0.38 ± 0.06	0.49 ± 0.09	0.46 ± 0.07	0.41 ± 0.06
	CHOL	mmol/L	1.89 ± 0.20	1.65 ± 0.08	1.96 ± 0.16	2.06 ± 0.21
	HDL	mmol/L	0.93 ± 0.09	1.09 ± 0.10	1.03 ± 0.10	0.96 ± 0.11
	LDL	mmol/L	0.56 ± 0.07	0.43 ± 0.04	0.49 ± 0.03	0.63 ± 0.05
	CK	U/L	248.80 ± 32.32	155.60 ± 24.76	165.40 ± 15.23	216.20 ± 54.06
	LDH	U/L	322.60 ± 11.34	328.20 ± 28.79	292.60 ± 8.62	331.80 ± 12.94
	GT	U/L	70.80 ± 7.36	75.60 ± 7.91	65.80 ± 5.37	58.60 ± 3.63
	ALP	U/L	174.40 ± 16.98^b^	297.60 ± 29.78^a^	262.40 ± 34.14^ab^	321.40 ± 45.90^a^
Group 4	ALT	U/L	16.60 ± 2.25	17.60 ± 1.21	17.20 ± 0.58	20.80 ± 1.85
	AST	U/L	95.20 ± 4.22	92.00 ± 6.99	96.60 ± 8.05	106.20 ± 11.27
	TP	g/L	65.22 ± 1.76^b^	74.72 ± 2.67^a^	74.44 ± 2.42^a^	72.58 ± 2.88a^b^
	ALB	g/L	22.16 ± 0.47	22.92 ± 1.02	23.52 ± 0.93	24.22 ± 0.97
	GLO	g/L	43.06 ± 2.12	51.80 ± 3.44	50.92 ± 3.32	48.36 ± 3.09
	Urea-N	mmol/L	5.45 ± 0.42^a^	6.05 ± 0.28^a^	5.82 ± 0.30^a^	3.29 ± 0.39^b^
	CRT	mmol/L	40.80 ± 2.06	43.00 ± 2.65	45.20 ± 2.42	48.00 ± 1.97
	GLU	mmol/L	3.13 ± 0.23b	3.57 ± 0.27b	3.34 ± 0.23b	4.48 ± 0.43a
	Calcium	mmol/L	2.51 ± 0.05	2.56 ± 0.07	2.51 ± 0.05	2.49 ± 0.05
	Phosphorus	mmol/L	2.50 ± 0.24	3.16 ± 0.16	2.89 ± 0.14	2.52 ± 0.17
	TRIG	mmol/L	0.31 ± 0.04^b^	0.44 ± 0.06^b^	0.70 ± 0.11^a^	0.49 ± 0.06^ab^
	CHOL	mmol/L	1.69 ± 0.03	1.80 ± 0.09	1.81 ± 0.10	1.96 ± 0.08
	HDL	mmol/L	0.86 ± 0.03	0.91 ± 0.05	0.90 ± 0.05	0.90 ± 0.05
	LDL	mmol/L	0.49 ± 0.01^b^	0.50 ± 0.03^b^	0.50 ± 0.05^b^	0.65 ± 0.06^a^
	CK	U/L	257.80 ± 37.83	197.40 ± 19.11	203.20 ± 23.26	230.40 ± 50.55
	LDH	U/L	273.60 ± 12.36	306.40 ± 20.60	318.20 ± 19.91	312.40 ± 42.69
	GT	U/L	61.20 ± 6.76	63.20 ± 5.78	60.60 ± 3.72	57.00 ± 4.64
	ALP	U/L	196.80 ± 43.42	282.80 ± 40.26	287.00 ± 39.51	343.40 ± 55.14

*Data in a row with different small letters represented a significant difference at a 5% level, while the same or no letters indicated no significant difference*.

### Serum immune factors

Commercial ELISA kits were used to determine the levels of immune factors in serum. The levels of immunoglobulin, interleukin, and interferon in different treatment groups are compared in Tables 6–**8**, respectively. Unlike serum biochemical factors, the time-dependent effect comparisons were not performed for these immune factors. Before the alfalfa saponins treatments, the levels of all tested immune factors were not significantly different among the four groups. However, after different feeding periods, the level of immunoglobulin increased to a certain extent compared with the control group (Table 6). Alfalfa saponins had no significant effect on IL-6 levels, and the impact on IL-1 was only observed in Groups 3 and 4 (Table 7). The levels of serum IFN-α and IFN-β in the alfalfa saponins-treated groups were also increased to a certain extent compared with the control group, but a similar effect on IFN-γ was not observed (Table 8).

**Table 6:** Comparison of serum immunoglobulin levels (mean ± SD) among four treatment groups (*n* = 5 in each group).

**Immune factors**	**Continuous feeding time (day)**	**Group 1**	**Group 2**	**Group 3**	**Group 4**
IgA (mg/mL)	0	6.34 ± 1.67	7.26 ± 1.71	5.38 ± 0.30	7.06 ± 0.64
	14	5.58 ± 0.98^b^	7.59 ± 1.45^ab^	9.68 ± 1.88^a^	8.97 ± 1.76^a^
	24	6.16 ± 1.15^b^	6.36 ± 1.04^b^	10.89 ± 0.33^a^	7.47 ± 1.12^b^
	40	5.75 ± 1.06^b^	6.14 ± 1.15^b^	9.63 ± 3.29^a^	6.89 ± 1.00^b^
IgG (mg/mL)	0	11.27 ± 0.63	10.58 ± 2.83	12.81 ± 0.71	10.76 ± 1.88
	14	10.68 ± 2.31^b^	10.51 ± 1.95^b^	14.39 ± 1.66^a^	15.96 ± 1.93^a^
	24	10.83 ± 1.05^b^	10.72 ± 1.00^b^	15.45 ± 1.30^a^	16.66 ± 1.22^a^
	40	11.64 ± 1.09^b^	11.86 ± 1.01^b^	16.30 ± 1.57^a^	17.02 ± 2.14^a^
IgE (μg/mL)	0	762.15 ± 45.23	698.65 ± 37.27	713.01 ± 38.24	748.61 ± 21.54
	14	838.99 ± 33.32	748.26 ± 52.74	801.33 ± 51.18	855.78 ± 12.21
	24	812.85 ± 77.34^b^	872.41 ± 49.74^ab^	888.33 ± 22.22^a^	934.73 ± 27.37^a^
	40	821.63 ± 24.14^b^	894.68 ± 30.94^ab^	983.33 ± 46.62^a^	947.02 ± 208.68^a^
IgM (mg/mL)	0	3.16 ± 0.07	3.96 ± 0.81	3.59 ± 0.13	3.70 ± 1.02
	14	3.89 ± 0.45^b^	4.23 ± 0.12^ab^	5.09 ± 0.94^a^	4.09 ± 0.73^ab^
	24	4.35 ± 0.38^b^	4.09 ± 0.19^b^	6.14 ± 2.99^a^	4.47 ± 0.23^b^
	40	3.43 ± 0.23^b^	3.15 ± 0.45^b^	7.57 ± 2.56^a^	5.38 ± 2.44^a^

*Data in a row with different small letters represented a significant difference at a 5% level, while the same or no letters indicated no significant difference*.

**Table 7:** Comparison of serum interleukins levels (mean ± SD) among four treatment groups (*n* = 5 in each group).

**Immune factors**	**Continuous feeding time (day)**	**Group 1**	**Group 2**	**Group 3**	**Group 4**
>IL-1 (pg/mL)	0	108.96 ± 15.76	117.08 ± 11.51	114.76 ± 27.87	128.82 ± 33.92
	14	127.98 ± 12.20^b^	122.22 ± 7.21^b^	124.64 ± 12.45^b^	153.29 ± 16.73^a^
	24	131.84 ± 24.99^b^	129.27 ± 20.86^b^	135.60 ± 20.25^b^	157.70 ± 25.96^a^
	40	128.96 ± 16.81^b^	116.36 ± 5.73^b^	142.39 ± 10.78^a^	158.67 ± 17.65^a^
>IL-6 (ng/mL)	0	353.87 ± 25.56	361.74 ± 24.82	378.98 ± 22.30	384.54 ± 24.48
	14	329.45 ± 22.53	355.25 ± 31.63	331.86 ± 34.01	351.36 ± 34.44
	24	360.77 ± 30.22	376.80 ± 32.21	385.97 ± 31.54	367.65 ± 15.35
	40	374.21 ± 28.09	359.21 ± 27.42	323.78 ± 28.12	343.23 ± 21.27

*Data in a row with different small letters represented a significant difference at a 5% level, while the same or no letters indicated no significant difference*.

**Table 8:** Comparison of serum interferons levels (mean ± SD) among four treatment groups (*n* = 5 in each group).

**Immune factors**	**Continuous feeding time (day)**	**Group 1**	**Group 2**	**Group 3**	**Group 4**
IFN-α (ng/L)	0	32.73 ± 2.65	34.05 ± 2.96	33.73 ± 3.47	31.61 ± 7.50
	14	38.35 ± 5.20^b^	40.33 ± 3.68^b^	38.12 ± 4.86^b^	63.50 ± 9.33^a^
	24	35.48 ± 4.72^c^	40.48 ± 3.38^b^	47.32 ± 4.02^b^	65.62 ± 3.53^a^
	40	37.10 ± 3.75^c^	46.30 ± 2.00^b^	58.31 ± 2.27^ab^	65.74 ± 7.85^a^
IFN-β (ng/L)	0	41.67 ± 2.24	38.59 ± 2.25	43.91 ± 3.32	42.27 ± 2.51
	14	34.87 ± 3.60^c^	37.09 ± 3.67^b^	45.81 ± 4.21^ab^	46.51 ± 3.88^a^
	24	38.63 ± 5.87^b^	36.03 ± 2.73^b^	53.67 ± 4.11^a^	47.12 ± 3.32^a^
	40	35.83 ± 2.09^c^	31.46 ± 2.26^c^	90.66 ± 0.76^a^	49.32 ± 3.57^b^
IFN-γ (ng/L)	0	262.67 ± 21.66	300.71 ± 17.15	363.22 ± 23.32	354.12 ± 1.98
	14	330.97 ± 10.88	326.02 ± 20.94	378.49 ± 19.83	315.31 ± 16.99
	24	287.06 ± 16.13	330.75 ± 13.87	357.43 ± 16.23	322.70 ± 9.61
	40	320.87 ± 28.05	317.74 ± 24.81	348.72 ± 24.66	340.18 ± 17.23

*Data in a row with different small letters represented a significant difference at a 5% level, while the same or no letters indicated no significant difference*.

## Discussion

The dose-dependent effects of alfalfa saponins on the growth performance of Small-Tailed Han sheep were observed in the current study. Small-Tailed Han sheep in the 10 g/kg supplemental dose group had the highest ADG (0.258 ± 0.071 kg/day), which was significantly higher (*P* < 0.05) in comparison to the control group and group receiving 20 g/kg saponins (0.090 ± 0.031 and 0.168 ± 0.072 kg/day, respectively). Initially, saponins were generally considered the main antinutritional factors in alfalfa because of their bloat effects in the rumen (7, 18). However, lately, several studies have shown the beneficial effects of alfalfa saponins (5, 9, 14, 19). Alfalfa saponins would reduce microbial fermentation and nutrient degradation in the rumen; however, the nutrient absorption was increased in the small intestine (18). The authors did not examine the species and abundances of microbes in the rumen in the present study; however, the sheep in the highest supplementation dose group (20 g/kg) had a lower ADG value than the 5 and 10 g/kg supplementation groups, also confirming the antinutritional properties of alfalfa saponins at relatively higher levels.

A previous study has demonstrated that alfalfa saponins could improve meat quality in Hu lambs (9), including bringing a bright red meat color, lowering intramuscular fat content, and decreasing the drip loss of muscle. Similar results were also found in the current Small-Tailed Han sheep. The drip loss, cooking loss, and shear force decreased in the alfalfa saponins treatment groups (Table 3). The existing results have confirmed that alfalfa saponins could improve the meat quality, especially by reducing the shear force and further improving the meat tenderness. In the previous study in Hu sheep (9), lower supplemental doses were used, ranging from 0.5 to 4 g/kg; however, a more extended feeding period was used, which lasted 90 days. Such an extended feeding period may be worth exploring further because the results on serum biochemical factors showed that the effect of long-term supplementation was not ideal. The sheep used in the current study were in the breeding stage, and their body weights were close to the slaughter weight, so long-term feeding experiments were not carried out. In the future, similar studies should be performed in Small-Tailed Han lambs to explore the optimal supplementation dose for lambs.

The levels of Urea-N and GLU are susceptible to many uncontrolled factors during feeding trials. The present results showed that alfalfa saponins might not affect the metabolism of protein and glucose in plasma with no differences in Urea-N and GLU levels. Cholesterol is known as a cell membrane stabilizer. Loss of cholesterol leads to membrane instability and membrane lysis. In the present study, the decrease in cholesterol after alfalfa saponins treatments was inconsistent with the previous results in Hu lambs (5), which might result from different supplementation doses, different feeding periods or different sheep breeds. In addition to the dose-dependent effects of alfalfa saponins, this study also compared the time-dependent effects of alfalfa saponins supplementation on the serum biochemical factor levels. Although the sheep in the control group had not been exposed to alfalfa saponins during the 40-day feeding period, as the sheep grew, the serum levels of Urea-N, GLU, CK, and LDH were all changed (Table 5). Excluding these changes in biochemical factors caused by the growth of sheep, the time-dependent effects were indeed observed in the treatment groups. In Group 2, with the prolongation of feeding time, the concentrations of ALB and CHOL showed a significant upward trend, while the contents of Urea-N and CK showed a significant downward trend (Table 5). In Group 3, a changing trend in Urea-N was consistent with Group 2; significant differences were also found in CRT and ALP, although no clear trends were found (Table 5). In Group 4, the concentration of TRIG first increased (reaching the peak concentration of 0.70 ± 0.11 mmol/L on Day 25) and then decreased with the prolongation of feeding time (Table 5). The influence of alfalfa meal on the serum biochemical profile in broilers has also been identified in a previous study (20). In that study, alfalfa meal was supplemented at four levels from 0 to 75 g/kg diet. And the serum levels of AST and CHOL were both decreased by the supplement of alfalfa meal. One previous study has confirmed that N intake and the type of N source can affect serum Urea-N concentrations (21). Additionally, a high correlation has been found in ruminants between serum Urea-N concentration and rumen ammonia concentration (21). However, the rumen ammonia levels were not determined in the present study. Therefore, the potential correlation could not be determined.

After continuous feeding of alfalfa saponins, almost all serum immunoglobulin levels were elevated compared with the control group. After 14 days of feeding, IgA, IgG, and IgM concentrations were significantly higher than those of the control group (Table 6). Similar results were also found for IL-1 (Table 7), IFN-α, and IFN-β (Table 8). Immunity can be divided into innate and acquired ones. The former is formed by the body innately and inherited from parents, while the acquired immunity is the immunity acquired in fighting against pathogenic microorganisms (22). Innate immunity mainly involves cytokines, such as immunoglobulin, interferon, and interleukin (14, 23). Currently, there is no research on the effect of alfalfa saponins on the immune function of ruminants. The researchers compared the effects of betaine and alfalfa saponins on IgA, IgG, and IgM in the serum of weaned piglets (24). They found that both compounds could increase the serum IgG levels, with alfalfa saponins being more effective than betaine. There are few studies on the effects of alfalfa saponins on immune function, instead of focusing on alfalfa meal or fermented alfalfa (25–28). These studies have confirmed the immune-enhancing effects of alfalfa, but whether these effects are derived from alfalfa saponins needs to be further explored.

## Conclusion

The present study has determined the potential effects of alfalfa saponins on the growth performance, meat quality, serum biochemical, and immune factors in Small-Tailed Han sheep. The results validated the effects of alfalfa saponins on the growth performance and meat quality in Small-Tailed Han sheep, and the supplementation level of the 10 g/kg diet was the best. Alfalfa saponins also had effects on the levels of biochemical factors in serum. Additionally, the dose- and time-dependent effects were observed. After a 14-day feeding period, the concentrations of cholesterol (CHOL) and low-density lipoprotein (LDL) in the three treatment groups were lower than those in the control group. But when continued feeding, the effect became less noticeable or even disappeared. Supplying alfalfa saponins increased serum concentrations of IgA, IgG, IgE, IgM, IL-1, IFN-α, and IFN-β. Based on the current results, the alfalfa saponins concentration of 10 g/kg diet (for 14 consecutive days) could be suggested as the optimum ratio for good health conditions of Small-Tailed Han sheep.

## Data availability statement

The raw data supporting the conclusions of this article will be made available by the authors, without unduereservation.

## Ethics statement

The animal study was reviewed and approved by the Institutional Animal Care and Use Committee (IACUC) of Henan University of Science and Technology.

## Author contributions

FanY and Y-QW conceived this project. FangY, Z-HZ, and S-QW performed animal experiments. LZ and B-LZ determined the serum immune factor levels. J-CC performed the serum biochemical factors analysis. FanY performed the statistical analysis and wrote this manuscript with support from FangY. All authors read and approved this final manuscript.

## Funding

This study was financially supported by the China Agriculture Research System of MOF and MARA (Grant No. CARS-38), the Natural Science Foundation of Henan (Grant No. 212300410037), and the Foundation for University Young Key Teacher Program of Henan Province (Grant No. 2021GGJS044).

## Conflict of interest

The authors declare that the research was conducted in the absence of any commercial or financial relationships that could be construed as a potential conflict of interest.

## Publisher's note

All claims expressed in this article are solely those of the authors and do not necessarily represent those of their affiliated organizations, or those of the publisher, the editors and the reviewers. Any product that may be evaluated in this article, or claim that may be made by its manufacturer, is not guaranteed or endorsed by the publisher.
